# An Intrathoracic Meningocele in a Neurofibromatosis Type I Patient Mimicking Severe COVID-19 Disease

**DOI:** 10.7759/cureus.29872

**Published:** 2022-10-03

**Authors:** Vasileios K Mousafeiris, Ioannis Papaioannou, Georgia Pantazidou, Nektaria Kalyva, Thomas Repantis

**Affiliations:** 1 Orthopedics, General Hospital of Patras, Patras, GRC; 2 Otolaryngology - Head and Neck Surgery, General Hospital of Patras, Patras, GRC; 3 Pediatrics, University Hospital of Patras, Patras, GRC

**Keywords:** immunosuppression, neurofibromatosis-1 (nf-1), differential diagnoses, covid 19, thoracic meningocele

## Abstract

Intrathoracic meningoceles (IM) are quite rare; they are commonly associated with neurofibromatosis type 1 (NF-1). We report a case of a 55-year-old lady who was admitted to our emergency department with a sore throat, mild fever, cough, and right-sided chest pain, and tested positive for coronavirus disease 2019 (COVID-19). Ιmaging revealed a meningocele in the right upper pulmonary area, attributed to her NF-1. Clinicians should be aware that patients with NF-1 can develop IM, and they should be included in the differential diagnosis of patients with an intrathoracic mass.

## Introduction

Neurofibromatosis type 1 (NF-1) is a genetically inherited, neurocutaneous disorder, affecting almost one individual per 3000 population [1]. Mutation of NF-1 causes alterations in inflammatory cell number and activity as well as changes in cytokines, suggesting that immune response is defective, leading to infections and even cancer [2]. Spinal meningocele is the protrusion of the dura mater and cerebrospinal fluid (CSF) through a bony defect. It is most common in the lumbosacral region but can occur anywhere along the spine. A single intrathoracic meningocele (IM) is rarely reported. Most of them are associated with syndromes. More specifically, 60% to 85 % of all IM are associated with NF-1 [3]. Phol et al. reported the first case of an IM in 1933 [4]. The clinical manifestations of IM have a wide spectrum, ranging from completely asymptomatic to mildly or more severely symptomatic. The majority of IM are asymptomatic and only regular follow-up, without intervention, should suffice [5]. In cases with remarkable IM, clinicians may notice cough, decreased respiratory sounds, abnormal respiratory functional tests, or dyspnea; neglected IM can even cause paraparesis [5,6]. On the other hand, coronavirus disease 2019 (COVID-19) infection itself can also cause cough, shortness of breath, and abnormal findings in respiratory tests [7]. We present a case of an NF-1 patient with a large intrathoracic mass, which was initially attributed to COVID-19 infection. To the best of our knowledge, this is the first case in which an IM was initially misdiagnosed as severe COVID-19 disease in an NF-1 patient.

## Case presentation

A 55-year-old lady, with a known history of NF-1 since the age of 22, was admitted to our emergency department complaining of sore throat, mild fever, and cough. Physical examination revealed widespread café-au-lait macules along with subcutaneous nodules, prominent bilateral freckling in the axillae, and diminished breath sounds in the upper right pulmonary area. A nasopharyngeal polymerase chain reaction (PCR) COVID test was obtained, which was positive and therefore the patient was admitted to the COVID-19 unit of our hospital. Laboratory blood tests revealed increased white blood cell (WBC) count, C-reactive protein (CRP), and erythrocyte sedimentation rate (ESR) while D-dimers, international normalized ratio (INR), prothrombin time (PT), partial thromboplastin time (PTT), and platelet count were within normal limits (Table 1).

**Table 1:** Laboratory work-up revealed increased WBC count, CRP, and ESR, consistent with infection WBC = white blood cell, CRP = C-reactive protein, ESR = erythrocyte sedimentation rate, INR = international normalized ratio, PT = prothrombin time, PTT = partial thromboplastin time

Parameters	Value	Normal Range
WBC count	12.5 k/Ml	4-10 k/Ml
CRP	1.2mg/dL	<0.3mg/dL
ESR	45mm/ 1st h	0-20 mm/ 1st h
Platelet count	317,000/ml	150,000-450,000
INR	1.0	0.8-1.1
PT	12.4 sec	11-13.5 sec
PTT	31	25-35 sec
D- Dimers	0.28 μ/mL	<0.50 μ/mL

**Figure 1:**
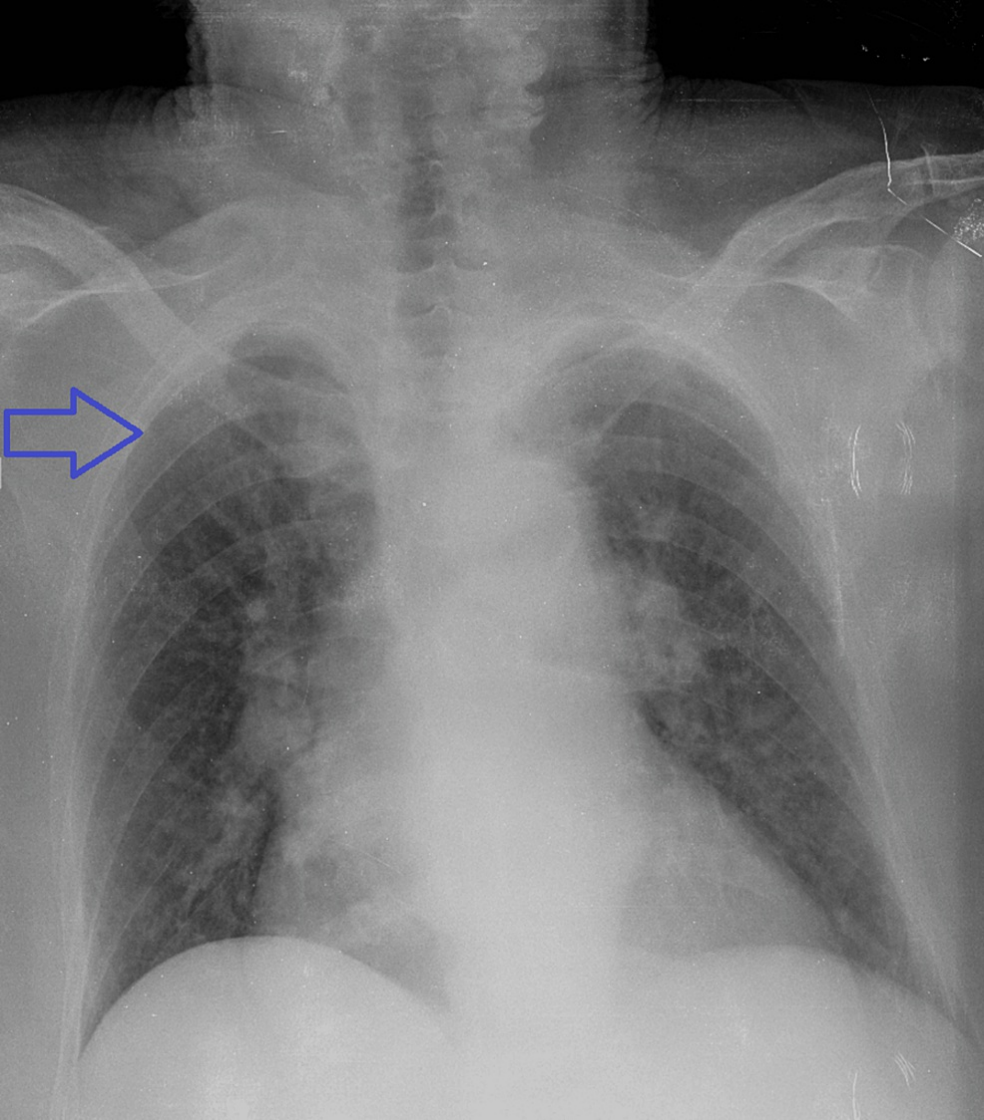
Chest X-ray displaying the area of diminished respiratory wheeze on the right upper pulmonary field (blue arrow)

Chest radiography showed a large mass-like lesion of the right lung (Figure 1).

**Figure 2:**
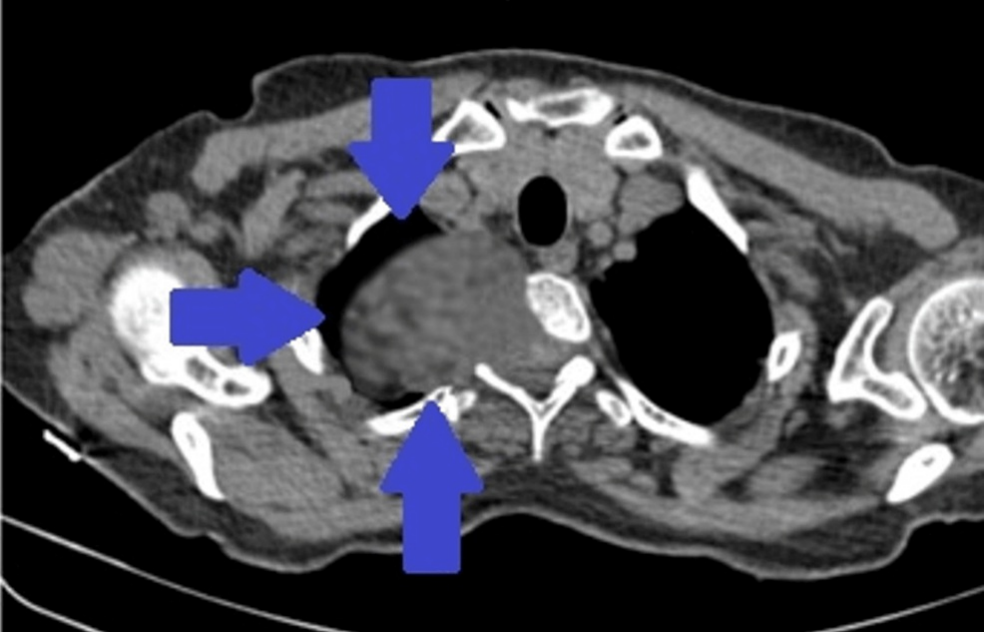
CT scan showing the large intrathoracic mass (blue arrows)

Based on clinical examination (respiratory symptoms) and initial chest X-ray imaging (mass-like lesion), the patient underwent a chest computer tomography (CT) for further investigation of this entity. Chest CT revealed a large mass that projected into the right hemithorax (Figure 2).

**Figure 3:**
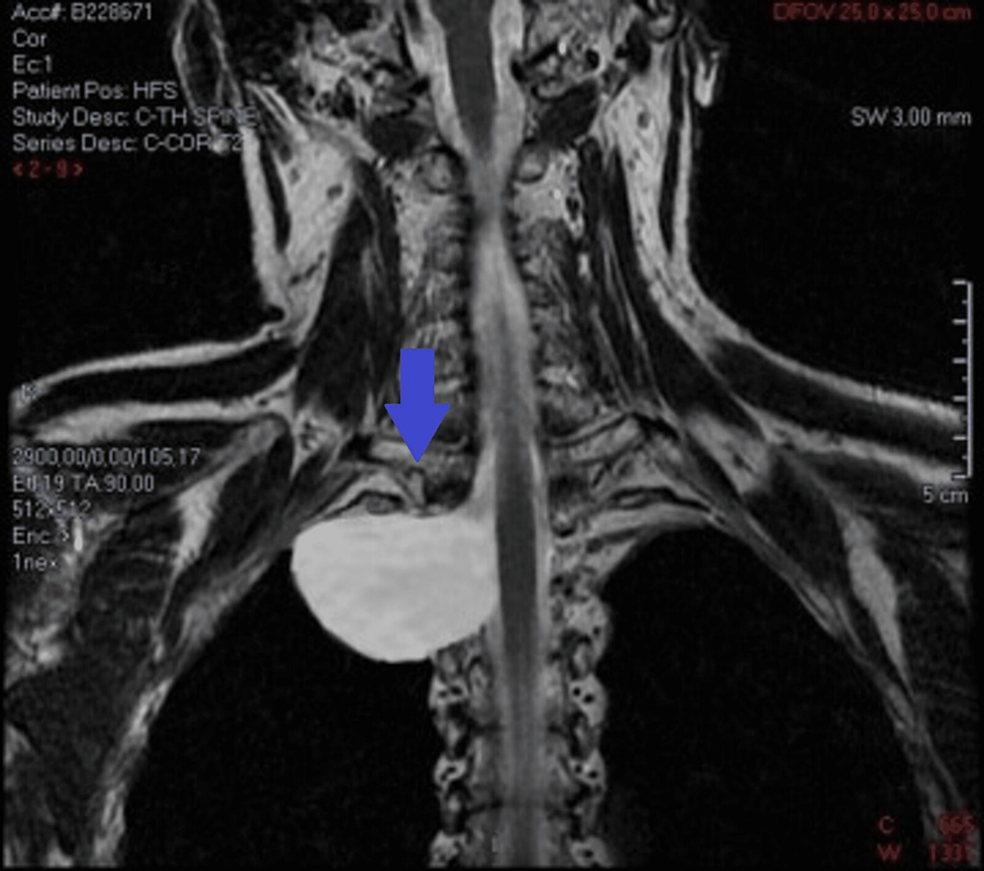
MRI showing the intrathoracic meningocele (blue arrow)

Magnetic resonance imaging (MRI) of the thoracic spine was subsequently performed to further clarify the origin of this mass and its extension to the surrounding tissues (Figure 3); the diagnosis of IM was confirmed.

The patient thereafter was referred for a multidisciplinary approach to a team of thoracic and spine surgeons along with close follow-up by pulmonologists.

## Discussion

To the best of our knowledge, this is the first report of a patient with IM due to NF-1 with a positive COVID-19 test that was initially considered a COVID-19 infection. It is important for clinicians to be aware that patients with NF-1 may more frequently (60-85%) present with symptoms from thoracic meningoceles and guide their management respectively [3].

A meningocele commonly occurs in the first two months of prenatal development as a sac-like protrusion of the spinal meninges through the vertebrae and can be associated with syndromes such as in NF-1 [1]. The clinical picture can vary and depends on the size, location, and number of IM, as well as the relationship of the IM with the surrounding tissues [8,9]. IM is generally small or asymptomatic, however, larger IM can cause dyspnea, chest or back pain, or neurologic symptoms such as paraparesis [8,9].

Meningoceles have been reported, although rarely, in any part of the body, and they are usually associated with NF-1 [10]. More specifically, Nanson et al. reported that 60% of IM were associated with NF-1 and scoliosis and that there is a three-fold association with syndromes [10]. Radiographically, meningoceles can be misdiagnosed initially as tumors, therefore extensive work-up is needed to exclude tumors arising mostly from the posterior mediastinum [11]. MRI plays an important role in the diagnosis, as it can visualize the meningocele well and provides information on its relationships with the surrounding tissues. The diagnosis of IM should be considered when the patient presents with multiple neurofibromas and kyphoscoliosis, and a mass is revealed in the posterior mediastinum in the MRI [11]. In our patient, multiple skin lesions were noted before the imaging workup revealed the right IM.

The management of IM ranges from observation with close follow-up to surgical excision and depends, among others, on the location, size, number of meningoceles, and relationship with the surrounding tissues. When the patient is asymptomatic, surgical treatment should be deferred; however, it is usually implemented for symptomatic growing lesions [12,13]. Single IM can be removed surgically, while in multiple lesions and in meningoceles with large dural connections, surgical excisions should be avoided [8]. Regular follow-up is recommended regardless of whether the meningocele was treated surgically or conservatively.

## Conclusions

Intrathoracic meningocele is relatively rare; however, in patients with NF-1, its prevalence is high. Clinicians should be, therefore, aware and include this rare entity in their differential diagnosis, as intrathoracic meningocele can present with respiratory symptoms and may go undiagnosed. Its treatment varies according to its characteristics and a multi-disciplinary approach from different specialties is considered beneficial for the patients.
